# Comparison of a qualitative immunochromatographic test with two quantitative serological assays for the detection of antibodies to *Leishmania infantum* in dogs

**DOI:** 10.1186/s13028-019-0473-1

**Published:** 2019-08-07

**Authors:** Sergio Villanueva-Saz, Asier Basurco, Víctor Martín, Antonio Fernández, Araceli Loste, María Teresa Verde

**Affiliations:** 1 Department of Pharmacology and Physiology, Veterinary Faculty, University of Zaragoza, Miguel Servet 177, 50013 Zaragoza, Spain; 2 Clinical Immunology Laboratory, Veterinary Faculty, University of Zaragoza, Miguel Servet 177, 50013 Zaragoza, Spain; 3 Department of Animal Pathology, Veterinary Faculty, University of Zaragoza, Miguel Servet 177, 50013 Zaragoza, Spain

**Keywords:** Diagnostic techniques and procedures, Immunoglobulins, Canine leishmaniosis

## Abstract

Canine leishmaniosis is a disease caused by *Leishmania infantum,* a vector-borne parasite. Due to the zoonotic potential of canine leishmaniosis, infected dogs must be identified. Serological assays are the most common methods for the detection of *L. infantum* infection in dogs used in veterinary practice. The aim of the study was to assess the performance of a rapid immunochromatographic test (*FAST*est LEISH^®^, MEGACOR Diagnostik) for the detection of specific antibodies to that of the *L. infantum* in dog sera. The results were simultaneously compared using a commercial brand of indirect immunofluorescence antibody test and an in-house enzyme-linked immunosorbent assay as references. Between the two reference tests, 232 serum samples out of 244, produced concordant results while 12 exhibited discordant results. Of the 232 concordant samples, 121 were classified as *L. infantum* seropositive, and 111 samples were previously classified as *L. infantum* seronegative by a combination of the reference assays. All samples that were seropositive by the reference tests were also positive according to the rapid test, and only one sample that was seronegative according to the two reference assays was positive according to the rapid test. Compared with the reference tests, the rapid test sensitivity was 100%, specificity was 99.1%, accuracy was 99.6%, Cohen’s kappa coefficient was 0.99, and the area under receiver operating characteristic curve was 0.995. The *FAST*est LEISH^®^ is a rapid, qualitative in-clinic test with high sensitivity and specificity.

**Electronic supplementary material:**

The online version of this article (10.1186/s13028-019-0473-1) contains supplementary material, which is available to authorized users.

## Findings

Canine leishmaniosis is zoonotic disease caused by *L. infantum,* a vector-borne protozoan parasite transmitted by phlebotomine sand flies under natural conditions. Clinical manifestations of infection may range from mild to absent to mild, several and even fatal disease. This variability is thought to be the result of cell-mediated immune response from the host which may be influenced by the genetic background of the dog [1]. Individual incubation times range from a few months to several years [2]. The detection of *L. infantum* antibodies indicates existing or past infections. High antibody levels are often associated with a high parasitic load and disease [3]. Conversely, low antibody levels in clinically normal dogs with a negative result on molecular and/or parasitological tests may indicate exposure or early stages of *Leishmania* infection [4]. Different confirmatory techniques were used and the diagnosis was based on the clinical manifestation and/or the laboratory abnormalities that were compatible with the disease as well as by the confirmation of *L. infantum* infection [5].

Serological assays, including enzyme-linked immunosorbent assay (ELISA), indirect immunofluorescence antibody test (IFAT), and rapid tests, are the most common methods for the detection of infection in dogs [6]. Rapid tests have been used as an important first step in diagnostic algorithms, enabling results to be obtained within a short time [7].

The present study evaluated the diagnostic performances, including the sensitivity (probability of a positive test result among those having the target condition), specificity (probability of a negative test result among those without the target condition), accuracy (the ability of a test to differentiate the patient and healthy cases correctly) and area the receiver operating characteristic curve (measure of the usefulness of a test showing the relationship between sensitivity and specificity) of a rapid qualitative immunochromatographic in dogs with different anti-*Leishmania* antibody levels and compared the results from a combination of two quantitative serological tests, IFAT and ELISA, as reference tests.

Blood samples from 244 dogs of different breeds, ages, and genders that were naturally exposed to *L. infantum* infection were included in the study from May 2017 to May 2018. These dogs presented at the Veterinary Teaching Hospital of the University of Zaragoza (Spain) for different diagnostic purposes, including the annual screening program for clinically healthy dogs, cases of suspected clinical leishmaniosis, the blood donor screening program and pre vaccination screening for *L. infantum* infection (Table 1). The serological status was recorded by a retrospective review of sample files through the laboratory database (Tables 2 and 3). Two aliquots of each obtained serum sample were stored at -20 °C until testing. Because samples were collected for the sole intention of determining a diagnosis, ethical approval was not needed. However, the owners were required to sign an informed consent.

**Table 1:** Details of dogs including purpose of diagnosis and signalment

	Description	Number of dogs (%)
Purpose of diagnosis	Annual screening program for clinically healthy dogs	78 (32.0)
	Cases of suspected clinical leishmaniosis	103 (42.2)
	Blood donor screening program	23 (9.4)
	Pre vaccination screening for *L. infantum* infection	40 (16.4)
Signalment		
Sex	Male	115 (47.1)
	Female	129 (52.9)
Age	12 months of age	68 (27.9)
	≥ 12 months of age and < 8 years of age	135 (55.3)
	≥ 8 years of age	41 (16.8)
Race	Purebred	94 (38.5)
	Mixed-bred	150 (61.5)

**Table 2:** Frequency of clinical signs in diseased dogs (n = 121, with IFAT and ELISA result)

Lymphadenomegaly	98 (80.1)
Cutaneous involvement	86 (71.1)
Weight loss	67 (55.4)
Anorexia	54 (44.6)
Exercise intolerance	38 (31.4)
Ocular involvement	25 (20.7)
Pale mucous membranes	19 (15.7)
Fever	15 (12.4)
Lameness	11 (9.1)
Vomiting, diarrhea	7 (5.8)
Polyuria and polydipsia	4 (3.3)
Muscle atrophy	3 (2.5)
Splenomegaly	2 (1.7)

**Table 3:** Frequency of hematologic and biochemical alterations in diseased dogs (n = 121, with IFAT and ELISA result)

Hematological parameters	
Anemia	67 (55.4)
Other hematological abnormalities	
Neutrophilia	17 (14.0)
Lymphopenia	22 (18.2)
Lymphocytosis	4 (3.3)
Thrombocytopenia	10 (8.3)
Biochemical parameters	
Renal azotemia	9 (7.4)
Hyperproteinemia with hypoalbuminemia and inverted albumin: globulin ratio	75 (62.0)
Urinalysis	
Proteinuria	43 (35.5)
Decreased urine specific gravity	36 (29.8)

The rapid test (*FAST*est Leish^®^, MEGACOR Diagnostik, Hörbranz, Austria) was performed following the instructions of the manufacturer. All tests were stored at room temperature and were performed as described in the instructions supplied with the test kit. This rapid test employs a combination of monoclonal antibodies conjugated with colloidal gold particles and recombinant *L. infantum* antigens bound to the solid phase of a nitrocellulose membrane that detect anti-*L. infantum* antibodies in whole blood, plasma or serum from the dog. The antibodies against *L. infantum* present in the sample react in the conjugate pad with mobile monoclonal antibodies to form antibody complexes. These antibody complexes migrate along the nitrocellulose membrane and bind to fixed *Leishmania* spp. antigens, producing clear pink-purple colored test line. Particles that do not bind to the conjugate continue their course along the membrane and pass through the control line with membrane-fixed control antibodies. The control line shows that the sample and reagents have been properly applied and migrated through the device. The buffer diluent facilitates the migration and promotes the binding of antibodies to antigens. Samples with a clear test and control line are classified as positive, and samples that show a control line are classified as negative. Prior to the study, technicians were trained to perform and interpret results. All tests were read by two laboratory technicians after 15 min. If discrepancies arose between results, a third observer participated. The examiners were blinded to the results of the quantitative serological tests.

The commercial IFAT (MegaFLUO LEISH test^®^, MEGACOR Diagnostik, Hörbranz, Austria) was performed as described in the instructions supplied with the test kit. This commercial IFAT has been employed in other comparative studies of diagnostic tests [8]. All samples were examined by two different investigators. They were blinded to the results of the other serological tests. Samples negative at 1:100 were considered negative and the endpoint titer of positive samples was determined by preparing serial twofold dilutions of the serum starting from the cut-off value (1:100).

As a second quantitative serological reference method, an in-house ELISA by *L. infantum* was performed on all serum samples as previously described, with some modifications [9]. The in-house ELISA test was performed by a different researcher who had no knowledge of the rapid test and IFAT results. The cut-off value was 30 ELISA unit (EU). Sera with an EU ≥ 200 were classified as high positive, with an EU ≥ 100 and < 200 as moderate positive, and with an EU > 30 and < 100 as low positive (Additional file 1).

Data obtained were checked for normal distribution using the Kolmogorov–Smirnov test (P > 0.05). A regression analysis between the two quantitative tests and Pearson´s correlation coefficients were calculated for all results that were concordant among the three serological tests. Statistical analyses were performed using IBM SPSS statistics software version 22 (SPSS Inc., Armonk, NY, USA). Because a single widely accepted reference standard for the diagnosis of *L. infantum* infection in dogs is not available, samples were classified based on results coinciding in both quantitative reference tests. Samples for which discordant results were obtained with the reference tests were not included in the study. Moreover, dogs that had previously been vaccinated with either of the two vaccines available in Spain for the prevention of canine leishmaniosis were also not included in this study.

Of the 244 sera analysed, 232 samples showed concordant results for IFAT and the in-house ELISA. The remaining 12 samples were considered discordant between reference tests, including 2 samples with a low positive result for the in-house ELISA but a negative result (antibody titer < 1:100) for the IFAT, as well as 10 samples with a positive result (antibody titer of 1:100) for the IFAT but negative result for the in-house ELISA. All discordant samples between the two reference tests were classified as seronegative by the rapid test. Among the 232 serum samples characterized, 121 samples were classified as seropositive for *L. infantum* infection and 111 samples were classified as seronegative for *L. infantum* infection (Table 4). Serum samples with different serum anti-*Leishmania* spp. antibody levels were selected for the evaluation (Table 5). All serum samples classified as positive by both reference assays were also positive in the *FAST*est LEISH^®^. Only one seronegative sample classified by the reference assays was discordant and showed a positive rapid test result. There were no additional freeze–thaw cycle or sample handing differences in testing events between the three tests.

**Table 4:** Comparison of the *FAST*est LEISH^®^ with IFAT and ELISA results for *Leishmania* spp. antibody detection

Number of samples	IFAT and ELISA combination			IFAT		ELISA		Total
	Concordant results between reference tests		Discordant results between reference tests	Positive	Negative	Positive	Negative	
	Positive	Negative						
*FAST*est LEISH^®^								
Positive	121	1	0	121	1	121	1	122
Negative	0	110	12	10	112	2	120	122
Total	121	111	12	131	113	123	121	244

**Table 5:** Number of positive samples and antibody levels (IFAT and ELISA)

IFAT	1:400	1:800	1:1600	1:3200	1:6400	> 1:6400
Number of samples	4	8	27	22	14	46

ELISA cutoff: 30 EU	Low positive (> 30 and < 100 EU)	Moderate positive (≥ 100 and < 200 EU)	High positive (≥ 200 EU)			
Number of samples	27	46	48			

**Fig. 1:**
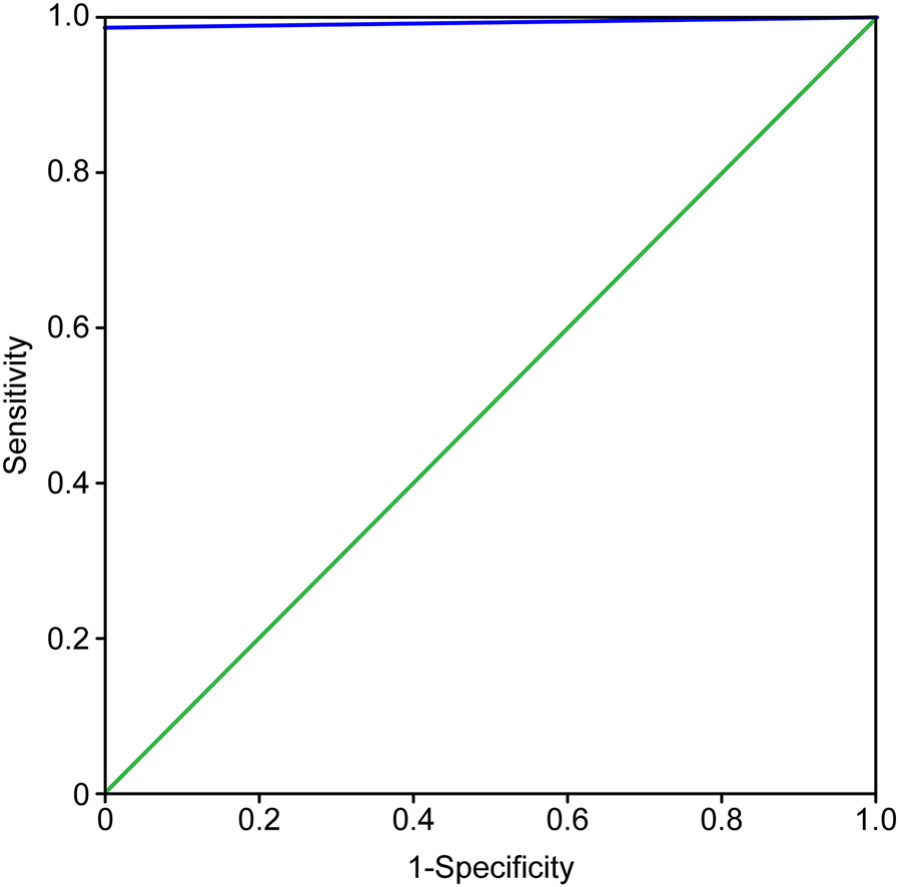
AUC-ROC curve analysis of the rapid test studied. The Area the receiver operating characteristic curve (AUC-ROC) analysis combines sensitivity and specificity into one measurement, and the result is a single global measure of diagnostic effectiveness. Each point on the ROC curve represents a sensitivity/specificity pair corresponding to a particular decision threshold. The blue line represents the AUC-ROC analysis of the *FAST*est LEISH^®^, and the green line represents the reference line

The rapid test achieved sensitivity of 100% (95% confidence intervals (CI) 96.2–100%) and specificity of 99.1% (95% CI 94.4–100%), and the accuracy was 99.6% (95% CI 97.3–100%). The Cohen’s kappa coefficient between the rapid test and the combined reference assays for the detection of antibodies was 0.99. The area the receiver operating characteristic curve (AUC-ROC) obtained from the curve was 0.995 (95% CI 0.985–1.000) (Fig. 1). Agreement between the two observers in reading the rapid test was 100% and no invalid results were obtained. The regression line was statistically significant (P < 0.001) and Pearson´s correlation coefficient was r = 0.675.

Sensitivity and specificity are common parameters for evaluating diagnostic methods [10]. Depending on the purpose of the test, sensitivity or specificity should be prioritized. When a test is used to detect *L. infantum* infection in dogs with suspected clinical leishmaniosis, specificity and sensitivity must be high, but importantly, specificity must not be too low because a confirmatory test should be highly specific to avoid false positives. In contrast, high sensitivity is essential for identifying clinically healthy infected dogs.

When a reliable gold standard is not available, at least two different tests should be performed [11]. In the present study, sample classification was established based on combination of results from ELISA and IFAT, obtaining a better definition of the serological status. When different serological tests are used simultaneously, discrepancies may be observed, and the lack of consistency among tests may depend on their different performances. A possible explanation for these discrepancies may be the inherent differences in the two quantitative assays. Therefore, the differences between the diagnostic performances of the references tests may modify the accuracy of the rapid test analyzed.

Numerous rapid tests are available to detect antibodies in dogs. However, these tests show high specificity and variable sensitivity [12]. Differences in diagnostic results obtained between rapid tests are influenced by the serum panel, technology, type of antigen, test threshold and the different laboratory test considered as a reference assay (Table 6). Standardization and comparison of results between the evaluated test and other similar rapid tests from different studies is not possible due to differences in the parameters used to evaluate a test, such as sample size, definition of the clinical status of the dog involved in the study and the selected reference populations. Therefore, examinations of diagnostic tests should follow the STARD guidelines (Standards for the Reporting of Diagnostic Accuracy Studies) to allow an adequate comparison between results.

**Table 6:** Concordant results between reference test and results of each reference test for sensitivity and specificity of the *FAST*est LEISH^®^

	Reference test		
	IFAT and ELISA combination	IFAT	ELISA
*FAST*est LEISH^®^			
Sensitivity (95% CI)	100% (96.2–100%)	92.4% (86.1–96.1%)	98.4% (93.7–99.8%)
Specificity (95% CI)	99.1% (94.4–100%)	99.1% (94.5–100%)	99.2% (94.9–100%)
Number of samples included	232	244	244

Sample inclusion can influence assessment measures with any test. Serum samples with high levels of anti-*Leishmania* spp. antibodies are more easily detected by a test and contribute to higher diagnostic values [13]. Samples in the present study had different antibody levels ranging from 42 to 372 EU, according to the in-house ELISA. Conversely, antibody titers detected by IFAT ranged from 1:400 to > 1:6400. The main differences between the ELISA and IFAT techniques are the type of antigen used and the technical method performed to obtain the results. In terms of interpreting the results, IFAT is subjective and depends on the operator´s experience, even when two different experienced observers examine the samples. This situation may explain the discordant results obtained.

A potential limitation of the present study was the lack of samples from dogs serologically reactive to other pathogens, particularly different *Leishmania* spp. or *Trypanosoma cruzi,* which are both protozoa that belong to the Trypanosomatidae family and share various antigens that may produce cross-reactions [14, 15]. All serum samples were collected from dogs from the city of Zaragoza, a region in Spain where *T. cruzi* is not present and *L. infantum* is the parasite responsible for canine leishmaniosis in Europe. Ideally, for a proper clinical sample characterization, the use of techniques of different nature is highly recommended. However, the objective of the study was to evaluated the ability of a rapid test to detect antibodies. For this reason and to characterize the seropositive status of the samples, serological techniques are necessary instead of other methods.

In conclusion, the use of *FAST*est LEISH^®^ as a screening test, may be particularly useful for identifying clinically healthy, seropositive, infected dogs.

## Additional file

**Additional file 1.** Description of the in-house indirect ELISA protocol for detection of anti-*Leishmania infantum* antibodies.

## Data Availability

The datasets used and/or analysed during the current study are available from the corresponding author on reasonable request.

## Abbreviations

AUC-ROC: area the receiver operating characteristic curve
CI: confidence intervals
IFAT: immunofluorescence antibody test
ELISA: enzyme-linked immunosorbent assay
EU: ELISA units
