# Supplementary material for: Comparison of a qualitative immunochromatographic test with two quantitative serological assays for the detection of antibodies to Leishmania infantum in dogs
Source: Acta Vet Scand. 2019 Aug 7;61:38. doi: 10.1186/s13028-019-0473-1 (PMC6686240; doi:10.1186/s13028-019-0473-1)
Supplement: Supplementary file 1 — Additional file 1. Description of the in-house indirect ELISA protocol for detection of anti-Leishmania infantum antibodies. [file 13028_2019_473_MOESM1_ESM.docx]

**Additional file 1.** “Description of the in-house indirect ELISA protocol for detection of anti-*Leishmania infantum* antibodies”

The *L. infantum* antigen (strain MHOM/FR/78/LEM 75 belonging to *Leishmania infantum* zimodeme MON-1) was provided by the Faculty of Pharmacy, University of Barcelona (Barcelona, Spain). Promastigotes were cultivated, and the parasite antigen was prepared. Promastigotes were grown in liquid Schneider’s medium supplemented with 20% fetal bovine serum (Thermo Fisher Scientific, Waltham, Massachusetts, USA) for 4 days at 26 °C. Stationary-phase promastigotes were washed four times with phosphate-buffered saline (PBS) and centrifuged. The packed promastigotes were resuspended in twice their volume of PBS and then sonicated in an ice solution. The disrupted parasites were collected, and the protein concentration was determined (BCA Protein Assay Kit, Thermo Fisher Scientific, Waltham, Massachusetts, USA). For the in-house ELISA, the crude antigen was adjusted to a concentration of 20 µg/mL with sterile PBS. Briefly, each plate was coated with 100 µL/well of the 20 µg/mL antigen solution in 0.1 M carbonate/bicarbonate buffer (pH 9.6) and incubated overnight at 4 °C. Plates were then frozen and stored at − 20 ºC. One hundred microliters of dog serum, diluted 1:800 in phosphate buffered saline (PBS) containing 0.05% Tween 20 (PBST) and 1% dry skimmed milk (PBST-M) were added to each well. The plates were incubated for 1 hour (h) at 37 °C in a moist chamber. After washing the plates three times with PBST for 3 minutes (min) followed by one wash with PBS for 1 min, 100 µL of Protein A conjugated to horseradish peroxidase (Thermo Fisher Scientific, Waltham, Massachusetts, USA) diluted 1:20000 in PBST-M was added to each well. The plates were incubated for 1 h at 37 °C in a moist chamber, followed by washes with PBST and PBS as described above. The substrate solution (ortho-phenylene-diamine) and stable peroxide substrate buffer (Thermo Fisher Scientific, Waltham, Massachusetts, USA) were added (100 µL per well) and developed for 20±5 min at room temperature in the dark. The reaction was terminated by adding 100 µL of 2.5 M H2SO4 to each well. Absorbance values were read at 492 nm (reference wavelength) in an automatic microELISA reader (ELISA Reader Labsystems Multiskan, Midland, Canada). Each plate included serum samples from a dog infected with *L. infantum* as confirmed by cytological examination as a positive control (calibrator) and serum samples from a healthy, noninfected dog from the blood donor program as a negative control. The same calibrator serum sample was used for all assays, and the plates with an interassay variation greater than 10% were discarded. All samples and controls were analyzed in duplicate. The results were quantified as ELISA units (EU) compared to a positive control serum sample used as a calibrator that was arbitrarily set to 100 EU.
